# The Application of a Three-Step Proteome Analysis for Identification of New Biomarkers of Pancreatic Cancer

**DOI:** 10.1155/2011/628787

**Published:** 2011-10-17

**Authors:** Mayinuer Abulaizi, Takeshi Tomonaga, Mamoru Satoh, Kazuyuki Sogawa, Kazuyuki Matsushita, Yoshio Kodera, Jurat Obul, Shigetsugu Takano, Hideyuki Yoshitomi, Masaru Miyazaki, Fumio Nomura

**Affiliations:** ^1^Department of Molecular Diagnosis, Graduate School of Medicine, Chiba University, Chiba 260-8670, Japan; ^2^Clinical Proteomics Research Center, Chiba University Hospital, Chiba 260-8677, Japan; ^3^Laboratory of Proteome Research, National Institute of Biomedical Innovation, Osaka 567-0085, Japan; ^4^Laboratory of Biomolecular Dynamics, Department of Physics, Kitasato University School of Science, Sagamihara 252-0373, Japan; ^5^Department of General Surgery, Graduate School of Medicine, Chiba University, 260-8670 Chiba, Japan

## Abstract

We searched for novel tumor markers of pancreatic cancer by three-step serum proteome analysis. Twelve serum abundant proteins were depleted using immunoaffinity columns followed by fractionation by reverse-phase high-performance liquid chromatography. Proteins in each fraction were separated by two-dimensional gel electrophoresis. Then the gel was stained by Coomassie Brilliant Blue. Protein spots in which the expression levels were significantly different between cancer and normal control were identified by LC-MS/MS. One hundred and two spots were upregulated, and 84 spots were downregulated in serum samples obtained from patients with pancreatic cancers, and 58 proteins were identified by mass spectrometry. These candidate proteins were validated using western blot analysis and enzyme-linked immunosorbent assay (ELISA). As a result of these validation process, we could confirm that the serum levels of apolipoprotein A-IV, vitamin D-binding protein, plasma retinol-binding protein 4, and tetranectin were significantly decreased in patients with pancreatic cancer.

## 1. Introduction

Pancreatic cancer is one of the most lethal malignancies, with a 5-year survival rate of only 4-5% [1]. The major reasons for the poor prognosis may be late diagnosis and limited therapeutic options; early diagnosis of pancreatic cancer is a pressing clinical problem.

Serum levels of the conventional tumor markers including carcinoembryonic antigen (CEA) and the Lewis blood group carbohydrate antigen (CA19-9) often remain in normal range at early stages of this malignancy [2]. Therefore, search for novel biomarkers of pancreatic cancer is needed.

Recent advances in proteomic technologies have provided promising ways to discover and identify novel biomarkers in various fields of clinical medicine. Although there has been long and uncertain path from marker discovery to clinical utility [3], sophisticated technologies have facilitated the discovery of potential tumor markers with improved sensitivities and specificities for the diagnosis of cancer patients [4]. Also, proteome analysis can lead to biomarkers that may be useful in the prediction of clinical response to anticancer therapy [5].

Surface enhanced laser desorption/ionization time-of-flight mass spectrometry (SELDI-TOF MS) is a representative example of a proteomics technique for the high-throughput fingerprinting of serum proteins and peptides and biomarker discovery [6]. Using this technology, we could detect and identify novel diagnostic markers for alcohol abuse [7] and also a new prognostic marker for pancreatic cancer [8].

One of the technical challenges in serum proteome analysis is that serum contains thousands of proteins and peptides that are present in a large dynamic concentration [9]. Indeed, 22 abundant proteins such as albumin, immunoglobulins, and transferring constitute up to 99% of the protein content of plasma [10]. In proteomic studies searching for low-abundance serum proteins or peptides, depletion of those abundant proteins and further fractionation of samples will be necessary.

We recently conducted a three-step proteome analysis involving removal of 12 abundant proteins and subsequent reversed-phase high-performance liquid chromatography fractionation and one-dimensional electrophoresis: we successfully identified three proteins including YKL-50 as a promising biomarker of sepsis [11].

Proteomics in pancreatic cancer research including serum or plasma biomarker search has been reviewed [12]. A three-step approach as-we used in this study has not been tried in biomarker search for pancreatic cancers before.

In this study, we applied this three-step proteome analysis to find novel biomarkers of pancreatic cancer.

## 2. Method

### 2.1. Patients Studied

Serum samples were obtained preoperatively from a total of 32 patients diagnosed with primary invasive pancreatic ductal carcinoma who had surgery at the Department of General Surgery, Chiba University Hospital. Clinical data of 32 patients are summarized in Table 1(a). Serum samples were also obtained from apparently healthy and age-matched subjects who had medical checkup at the Port-square Kashiwado Clinic, Kashiwado Memorial Foundation (Table 1(b)). All samples were frozen by liquid nitrogen and were stored at −80°C until analysis. Written informed consent was obtained from all the subjects. The ethics committee of our institute approved the protocol.

### 2.2. A Three-Step Serum Proteome Analysis

#### 2.2.1. Immunoaffinity Subtraction of Highly Abundant Proteins from Human Serum

Serum samples obtained from 4 patients with pancreatic cancer (Nos 1~4 in Table 1(a)) were pooled. Sera obtained from 4 age-matched healthy volunteers were also pooled (Nos 1~4 in Table 1(b)). As the first step of proteome analysis, the twelve most abundant proteins (albumin, Immunoglobulin G, transferrin, fibrinogen, Immunoglobulin A, Immunoglobulin M, apolipoprotein A-I, apolipoprotein A-II, haptoglobin, *α*1-acid glycoprotein and *α*2-macroglobulin) were removed from serum by passage through a commercially available immunoaffinity column, the ProteomeLab IgY12HC LC10 (Beckman coulter, Inc. Fullerton, CA. USA.) Ninety microliters of each pooled sample was subjected to the immunoaffinity subtraction as we previously described [11]. The combined flow-through fractions were concentrated by Vivaspin2 spin concentrators (molecular weight cutoff, 10 kDa, Vivascience, Hannover, Germany) and were stored at −80°C until use.

In addition, sera from 32 patients with pancreatic cancer and 32 healthy volunteers were used for validation. Eight healthy controls and 8 of relatively advanced cases (Nos 1~8 in Tables 1(a) and 1(b)) were chosen for first validation and 24 of them (Nos 9~32 in Tables 1(a) and 1(b)) were used for the second validation experiment.

### 2.3. HPLC Separation of Immunoaffinity-Subtracted Serum Samples

Immunoaffinity-subtracted serum samples prepared as described above were separated by reverse-phase HPLC in an automated HPLC system, the SHISEIDO Nanospace SI-2 (Shiseido Fine Chemicals, Tokyo, Japan) essentially as we described before [11]. A total of 40 fractions were collected at 0.5 min intervals from 19.6 to 39.6 min. Each fraction was immediately lyophilized by a centrifugal vacuum concentrator and stored at −80°C until further analysis.

### 2.4. Two-Dimensional Gel Electrophoresis

The IEF gels (70 mm length, Inner 2.5 mm diameter and pH ranges from 3 to 10) were prepared as previously described [13, 14]. The lyophilized samples (from fraction 6 to fraction 25) were dissolved with 15 *μ*L sample preparation buffer and proteins were separated by two-dimensional gel electrophoresis with agarose gels in the first dimension as described by Oh-Ishi et al. [13]. 

### 2.5. In-Gel Digestion and LC-MS/MS

CBB stained 2-DE images of pooled serum samples obtained from patients with pancreatic cancer were compared with those obtained from healthy volunteers. Differentially expressed protein bands were excised from the gel and were subjected to in-gel tryptic digestion as previously reported [14]. Digested peptides were injected into a trap column: 0.3 × 5 mm L-trap column (Chemicals Evaluation and Research Institute, Saitama, Japan) and an analytical column: 0.1 × 50 mm Monolith column (AMR, Tokyo, Japan), which was attached to a HPLC system (Nanospace SI-2; Shiseido Fine Chemicals, Tokyo, Japan). The flow rate of a mobile phase was 1 *μ*L/min. The solvent composition of the mobile phase was programmed to change in 35 min cycles with varying mixing ratios of solvent A (2% v/v CH_3_CN and 0.1% v/v HCOOH) to solvent B (90% v/v CH_3_CN and 0.1% v/v HCOOH): 5–50% B 20 min, 50–95% B 1 min, 95% B 3 min, 95–5% B 1 min, 5% B 10 min. Purified peptides were introduced from HPLC to an LTQ-XL (Thermo Scientific, CA, USA), an ion trap mass spectrometer (ITMS), via an attached Pico Tip (New Objective, MA, USA). The MS and MS/MS peptide spectra were measured in a data-dependent manner according to the manufacturer's operating specifications. The Mascot search engine (Matrixscience, London, UK) was used to identify proteins from the mass and tandem mass spectra of peptides. Peptide mass data were matched by searching the Human International Protein Index database (IPI, July 2008, 72079 entries, European Bioinformatics Institute) using the MASCOT engine. The minimum criterion of the probability-based MASCOT/MOWSE score was set with 5% as the significant threshold level. When the candidates had SEQUEST scores lower than 100 or when the SEQEUST score was computed by using fewer than one peptides fragment, we inspected the raw MS and MS/MS spectra of peptides to judge their qualities (see Figures a–f in Supplementary Material available online at doi: 10.1155/2011/628787). 

### 2.6. Western Blot Analysis

Western blotting was performed as we previously described [15].

Briefly, immunoaffinity-subtracted serum samples were separated on SDS-PAGE in 10–20% polyacrylamide gradient gel (DRC, Tokyo, Japan) and were transferred to polyvinylidene fluoride membranes (0.45 *μ*m thickness, Millipore, Bedford, MA) at 10 V for overnight. The following antibodies commercially available were used as primary antibodies; mouse anti-human ApoA-IV antibody (BML Inc., Tokyo, Japan), mouse anti-human GC antibody (LifeSpan, Inc., UK), mouse monoclonal anti-human RBP4 antibody (Abnova.Com., Taipei, Taiwan) and mouse anti-human CLEC3B antibody (BioPorto, Grusbakken 8, DK-2820 Gentofte, Denmark). Antigens on the membrane were detected with enhanced chemiluminescence detection reagents (GE Healthcare). Band intensities of the western blot images were quantified by TotalLab TL12 imaging analysis software (Shimadzu Co., Ltd. Kyoto, Japan) and were presented by arbitrary units.

### 2.7. Other Procedures

In addition to western blotting, ELISA was conducted in some marker candidates using human ApoA-IV ELISA kit (Millipore, Missouri, USA), GC ELISA kit (immundiagnostik AG, Bensheim), and RBP4 ELISA kit; (R & D systems). Their optical density was measured at 450 nm using a microplate reader (iMark Microplate Reader S/N 10288). Serum levels of CEA and CA19-9 were determined by established commercially available kits.

### 2.8. Statistical Analysis

Statistical analysis was conducted using KaleidaGraph 4.0 J (Synergy Software, Reading, PA) and IBM SPSS Statistics 18 (SPSS Inc., IL, USA). Significance was defined as *P* < 0.05. 

## 3. Results

### 3.1. Discovery and Identification of Differentially Expressed Proteins by a Three-Step Proteome Analysis

**Figure 1:**
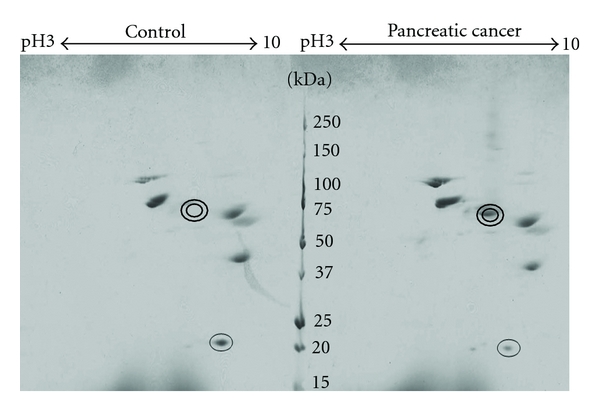
Comparison of 2-DE images of the same fraction of healthy volunteer control and pancreatic cancer patient sample. Electrophoresis was performed on the same gel and at the same condition. Figure 1 is an example of Coomassie blue-stained gel displaying spots from depleted and fractionated serum from control (left, *n* = 4, pooled) and pancreatic cancer patients (right, *n* = 4, pooled). (Fraction number is 10th). Double circles indicate increased spot in pancreatic cancer. Single circle indicates decreased spot in pancreatic cancer.

To discover and identify novel serum markers for pancreatic cancer, we employed a comparative three-step proteome analysis of the pooled serum samples obtained from patients with pancreatic cancer and healthy volunteers. As the first step, 12 abundant proteins were removed by immunosubtractions. The immunoaffinity-subtracted samples were separated by RP-HPLC, and 20 fractions (fractions Nos 6–25) were subjected to 2-DE. A representative example is shown in Figure 1. By comparing the 2-DE images of the proteins included in the 20 fractions, a total 186 spots were found to be differently expressed. Subsequent LC-MS/MS could identify 100 proteins. Excluding keratins, complements and trypsin, 58 proteins were selected; 37 of them were upregulated and 21 were downregulated (Tables 2(a) and 2(b)).

### 3.2. Validation of Marker Candidates by Western Blotting

Out of the 58 proteins listed in Tables 2(a) and 2(b), we focused on 19 proteins the alterations of which at serum level have not been studied in detail before, and also antibodies to be used for validation studies are available. Initial validation was conducted using 8 serum samples (nos. 1–8 in Table 1) obtained from relatively advanced cases with pancreatic cancer including the four cases used for the three-step analysis. Western blotting of the 19 proteins indicated in Tables 2(a) and 2(b) revealed that expression of 7 proteins were found to be significantly decreased in patients with pancreatic cancers compared with controls: they were inter-alpha trypsin inhibitor heavy chain H1 (ITIH1), hemopexin precursor (HPX), alpha-1B-glycoprotein precursor (A1BG), apolipoprotein A-IV precursor (ApoA-IV), vitamin D-binding protein precursor (GC), plasma retinol-binding protein precursor (RBP4), and tetranectin (CLEC3B).

**Figure 2:**
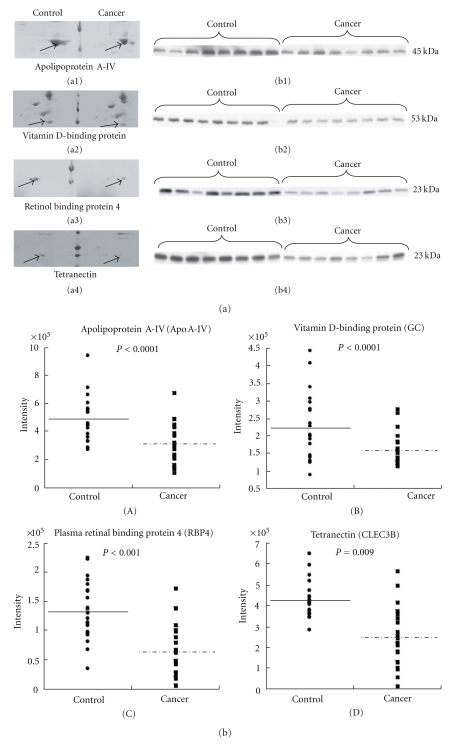
(a) Magnified views of 2-D gel images and western blotting analysis of ApoA-IV, GC, plasma retinol binding protein 4 RBP4, and CLEC3B in serum samples. Coomassie blue-stained 2-D gel images from pooled control and pancreatic cancer displaying the protein spots for ApoA-IV, GC, RBP4, and CLEC3B are shown in left panels (a1), (a2), (a3) and (a4). Western blotting of these four proteins are shown in the right panel (b1), (b2), (b3) and (b4). (b) Quantitation of differentially expressed serum proteins in pancreatic cancer and healthy volunteers by Western blot analysis. Intensities of each band were calculated by TotalLab TL 120 software. Closed circles indicate healthy volunteers and closed squares indicate patients with pancreatic cancer. Significance of the differences were calculated by using Wilcoxon Mann-Whitney test. Panel A: ApoA-IV levels of serum were significantly lower in the depleted sera of pancreatic cancer when compared with the depleted sera of healthy volunteers (*P* < 0.0001). Panel (B, C and D) are for proteins GC, RBP4, and CLEC3B and their serum levels were likewise lower in the pancreatic cancer patients. Their *P* values are lower than 0.0001, 0.001, and 0.009, respectively.

We then conducted the second validation study to test whether differential expression of the 7 protein candidates described above is reproducible using another set of serum samples obtained from 24 patients with pancreatic cancers including cases with relatively early stages (nos. 9–32 in Table 1(a)). As shown in Figure 2(a) the expression levels of the four proteins ApoA-IV, GC, RBP4, and CLEC3B were greater in cancer patients than in controls. The differences were statistically significant assessed by densitometry Figure 2(b).

### 3.3. Validation of Marker Candidates by ELISA

**Figure 3:**
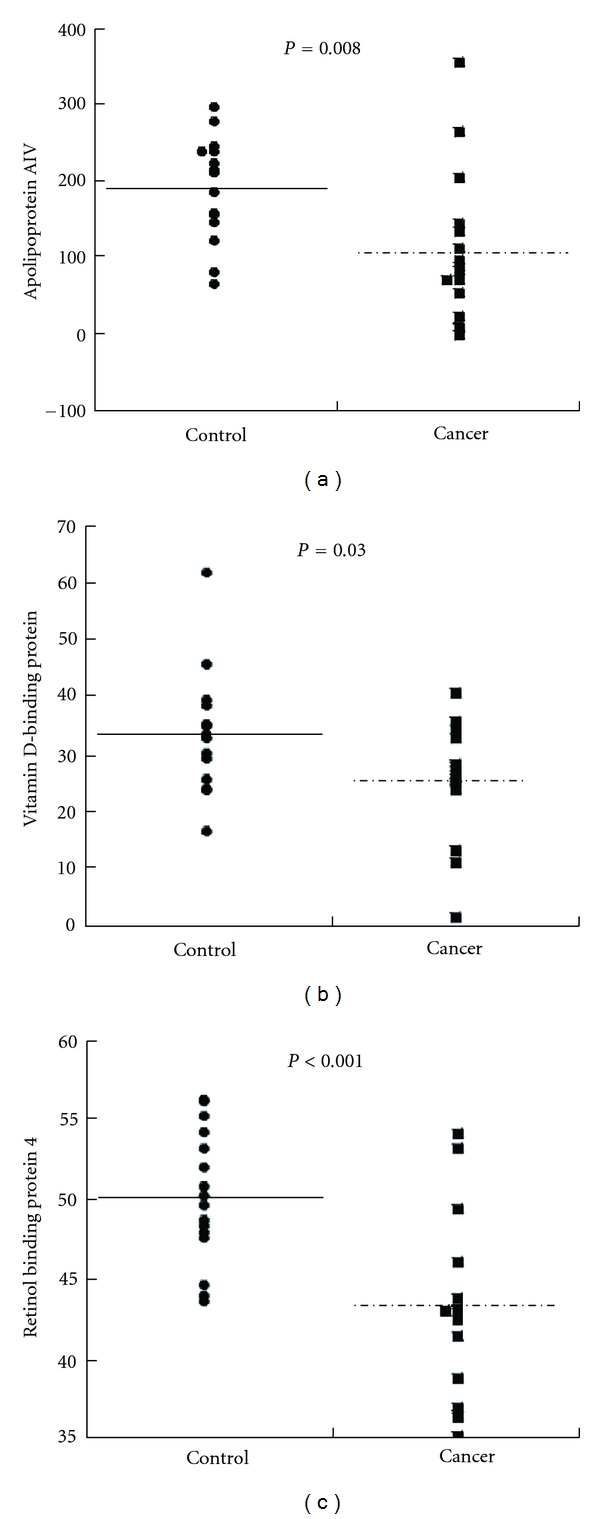
Quantitation of differentially expressed proteins in pancreatic cancer by ELISA. ELISA was performed using human ApoA-IV ELISA kit vitamin D binding protein ELISA kit and RBP4 ELISA kit, in serum samples obtained from 15 patients with pancreatic cancers and 15 control subjects. Analysis was performed by using Wilcoxon Mann-Whitney test. Closed circles indicate control and closed squares indicate cancer. (a): ApoA-IV levels of serum of patient with pancreatic cancer group (107.76 ± 25.8 AU) were lower than those in healthy group (185.27 ± 16.0 AU, *P* = 0.01); (b): GC levels were significantly lower in the patient group with pancreatic cancer (25.35 ± 9.8 UA) when compared with healthy group (34.40 ± 10.2 AU, *P* = 0.03); (c): RBP4 levels were lower in the pancreatic cancer group (42.99 ± 1.5 AU) than healthy group (50.7 ± 1.00 AU, *P* < 0.001).

ELISA kits were commercially available for GC, ApoA-IV, and RBP4. Their serum levels were determined in the 15 pairs of serum samples obtained from relatively early stages of patients used for the second validation by western blotting. Serum ApoA-IV levels of patients with pancreatic cancer (107.8 ± 99.9 AU) were significantly lower than those in healthy volunteers (195.2 ± 66.9 AU, *P* = 0.008) Figure 3(a). GC levels were significantly lower in the patient group with pancreatic cancer (25.4 ± 10 UA) when compared with healthy group (34.3 ± 10.3 AU, *P* = 0.03) Figure 3(b). Also, serum RBP levels in the patients (43.0 ± 5.9 AU) were significantly lower than in the controls (50.2 ± 4.2, *P* = 0.0004) Figure 3(c). 

### 3.4. Comparison of the Marker Candidates with CEA and CA19-9

**Figure 4:**
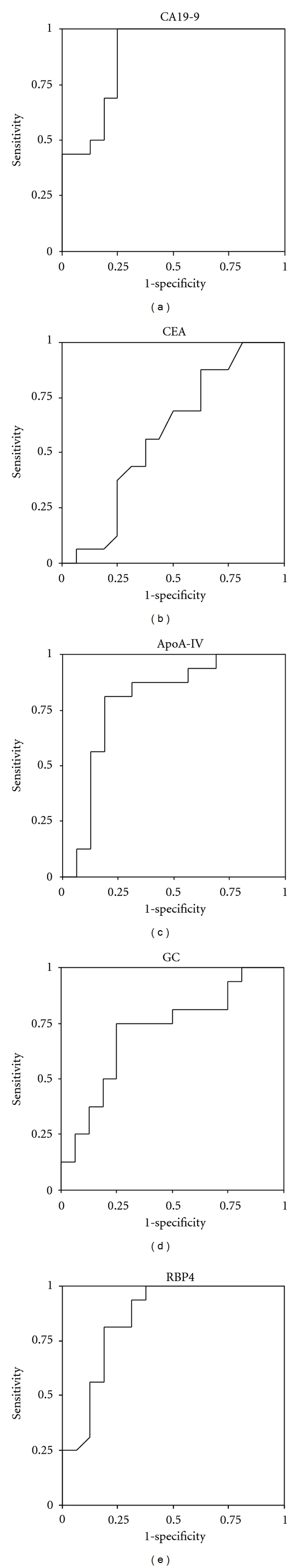
Receiver operating characteristic (ROC) curves for CA19-9, CEA, ApoA-IV, GC, RBP4 Their AUCs are described in the text.

Figure 4 shows the receiver-operating characteristic curve (ROC) analysis for the three marker candidates determined by ELISA and those for CEA and CA19-9.The areas under the curves for ApoA-IV, GC, RBP4, CA19-9, and CEA were 0.79, 0.72, 0.85, 0.88, 0.58, 0.88, 0.89, and 0.89, respectively. Also, AUCs of the combination assay of GC/CA19-9, ApoA-IV/CA19-9, and RBP/CA19-9 were 0.88, 0.89, and 0.89, respectively.

In Tables 3(a) and 3(b), serum levels of ApoA-IV, GC and RBP4 determined by ELISA are listed together with CEA and CA19-9. There were 7 cases in which serum CA19-9 level was not elevated. Out of these 7 cases, ApoA-IV levels were below the lower reference interval value (mean SD) in 2 cases. Also, GC levels were below the lower reference interval value in one case.

## 4. Discussion

The sequencing of the human genome has opened the door for comprehensive analysis of all the messenger RNA (transcriptome) and proteins (proteome). Messenger RNA concentrations, however, are not necessarily predictive of corresponding protein concentrations. Indeed, a recent report indicates that the sharing rate between cDNA microarray and proteome-based profilings is limited for the identification of candidate biomarkers in renal cell carcinoma [16]. Therefore, proteome analysis is one of the prerequisite for development of novel biomarkers. Proteomic studies in pancreatic cancers have been conducted by many research groups as reviewed [12, 17]. Hwang et al. found by using 2-DE/MS that phosphoglycerate kinase (PGK) 1, a secretable glycolytic enzyme involved in angiogenesis, is overexpressed in serum samples of pancreatic cancer patients, as compared to controls [18]. More recently, using the two-dimensional image-converted analysis of liquid chromatography and mass spectrometry (2DICAL) and a ‘‘glyco capturing” through concanavalin A-agarose, Ono et al. identified a novel prolyl-hydroxylation of fibrinogen alpha chain in plasma samples obtained from patients with pancreatic cancers [19].

**Figure 5:**
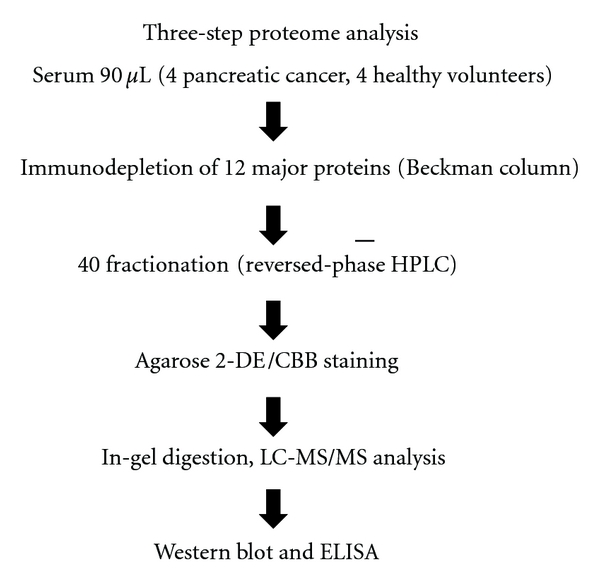
Outline of the procedure for the three-step serum proteome analysis.

In this study, the three-step procedure was carried out to discover novel markers of pancreatic cancer. The outline of the three-step procedures is shown in Figure 5.

As a first step, serum samples were subjected to antibody-based immunoaffinity column that simultaneously removes 12 abundant serum proteins. The concentrated flow-through was then fractionated using reversed-phase HPLC. Proteins obtained in each HPLC fraction were further separated by 2-DE. A total of 58 differentially expressed proteins were identified. As results of initial validation by western blotting in relatively advanced cases and further validation including the less advanced cases by western blotting, the expression levels of the four proteins ApoA-IV, GC, RBP4, and CLEC3B were greater in cancer patients than in controls. Out of these four proteins, ELISA were available in apolipoprotein A-IV, retinol-binding protein precursor (RBP4), and vitamin D binding protein (GC). Serum levels of these 3 proteins were significantly lower in patients with pancreatic cancer than in healthy volunteers. In ROC analyses, the area under the curves for these three proteins was not significantly greater than that for CA19-9, but it is noteworthy that among the 4 cases of pancreatic cancers in which serum levels of both CEA and CA19-9 were within the reference intervals, at least one of ApoA-IV, RBP4, and GC was found to be decreased in 2 cases, suggesting that these candidate markers could be complementary to the conventional markers in diagnosis of pancreatic cancer.

ApoA-IV is present in human intestinal epithelial cells and is secreted as a chylomicron and VLDL apoprotein [20].

Retinol binding protein 4 (RBP4) is a 21-kDa protein synthesized in the liver and adipose tissue; its major function is to deliver retinol to tissue [21]. Fabris et al. determined serum RBP levels in patients with pancreatic cancer and found that the levels decreased concomitant with zinc and prealbumin levels [22]. Serum zinc levels were not significantly correlated with RBP4 levels in the present study (data not shown).

Vitamin D-binding protein is a plasma protein involved in vitamin D transport and other function. Although diagnostic role of this protein in pancreas cancer has not been reported yet, inhibitory role of vitamin D binding protein-macrophage activating factor (DBP-maf) in pancreatic carcinogenesis has been pointed out [23].

Tetranectin binds to kringle 4 of plasminogen, enhancing the plasminogen activation by tissue-type plasminogen activator in the presence of poly-D-lysine [24] Low serum levels of tetranectin (CLEC3B) are associated with increased risk of second-line chemoresistance in patients with ovarian cancer [25]. Also, in colorectal cancer, significantly shorter survival was found for patients with CLEC3B levels below a cut-off point of compared to patients with levels above [26].

Thus, the results of this study show that four serum proteins, apolipoprotein A-IV, vitamin D binding protein, retinol-binding protein 4, and tetranectin are significantly decreased in patients with pancreatic cancer. It was notable that these changes were observed in some patients in whom conventional tumor markers for this malignancy were not altered.

The reasons why serum levels of these proteins were decreased in pancreatic cancer patients are not clear at the moment. It is unlikely that the alterations were entirely due to malnutrition because serum levels of the 4 proteins were not significantly correlated with their serum albumin levels. It is possible that some negative mediators originated from tumor and/or the cancer-tissue microenvironments were regulating their production. It is unlikely that the alterations were due to biliary obstruction because the extent of the alterations of the four markers were not related to the extent of biliary obstruction (data not shown). Alterations of these four proteins in chronic pancreatitis as well as biliary tract diseases remain to be studied. Also, it remains to be determined whether serum levels of these four proteins are changed in other gastroenterological cancers.

Although exact mechanisms responsible for the reduction remain to be investigated, alterations of serum levels of apolipoprotein A-IV, vitamin D binding protein, tetranectin, and retinol binding protein may have complementary role in diagnosis of pancreas cancer.

## Supplementary Material

Supplemental Figures: Raw data of the proteins which had SEQUEST scores lower than 100 or when the SEQEUST score was computed by using fever than one peptides fragment. The horizontal axis is molecular weight of peptide and the y-axis is intensity. Figure a: Raw date of isoform 1 of flcolln-3-precursor. Figure b: corticosteroid-binding globulin precursor. Figure c: cholinesterase precursor. Figure d: AMBP protein precursor. Figure e: tetranectin precursor. Figure f: histone 4.

## Figures and Tables

**Table 1a:** (a) Clinical features of pancreatic cancer patients.

No	Gender	Age (years)	UICC-stage	Tumor size (mm)	TP (g/dL)	ALB (g/dL)	Che (U/L)	T-Cho (mg/dL)
1	M	66	III	35	6.7	4.3	270	220
2	M	66	III	39	6.6	4.4	359	198
3	M	79	IV	10	6.2	3.8	179	155
4	M	71	III	18	6.8	4.0	207	210
5	M	65	III	35	7.0	3.7	189	131
6	M	78	III	50	6.6	4.4	359	198
7	M	66	IIA	30	6.3	4.1	267	176
8	M	62	IV	10	5.9	3.6	171	169
9	M	38	IA	10	6.7	4.0	289	179
10	M	50	IB	30	7.3	4.6	408	203
11	M	63	IIA	18	6.6	3.9	260	138
12	M	62	IIA	38	6.0	3.4	124	149
13	M	54	IIA	24	7.0	4.5	198	162
14	M	73	IIA	25	6.7	4.1	242	176
15	F	76	IIA	26	5.0	3.3	162	95
16	M	63	IIB	15	7.1	4.2	175	163
17	M	65	IIB	32	5.8	3.5	227	113
18	M	68	IIB	80	6.8	4.0	316	217
19	M	71	IIB	24	7.5	4.1	221	144
20	M	74	IIB	27	7.0	4.3	319	178
21	F	68	IIB	27	6.5	4.0	223	177
22	M	63	IIB	26	6.9	4.3	262	219
23	F	68	IIB	27	6.1	3.5	339	163
24	M	61	IIB	30	6.2	4.0	200	176
25	F	74	IIB	40	6.6	3.8	221	170
26	M	62	IIB	60	8.3	3.5	140	155
27	F	73	IIB	35	5.9	3.2	130	169
28	F	59	IIB	18	7.2	4.4	356	130
29	M	73	IIB	50	5.7	3.3	127	135
30	F	62	IIB	25	6.8	4.1	255	293
31	F	71	III	25	6.8	4.2	294	159
32	F	78	III	50	5.9	3.3	197	182

Ave ± SD		66 ± 8.6		30.9 ± 15.0	6.6 ± 0.6	3.9 ± 0.4	240.2 ± 75.6	171.9 ± 6.6

UICC: international union against cancer, M: male, F: female. TP: total protein. ALB: albumin. Che: cholinesterase. T-Cho: total cholesterol. Ave: average. SD: standard deviation.

From number 1 to 4 were used for 2-DE, from number 1 to 8 were for first western blot, from number 9 to 32 were for second western blot.

**Table 1b:** (b) Clinical features of healthy controls.

No	Gender	Age (years)	TP (g/dL)	ALB (g/dL)	Che (U/L)	T-Cho (mg/dL)
1	M	62	7.5	4.7	401	203
2	M	61	7.7	5.1	396	269
3	M	64	7.2	4.4	284	229
4	M	73	7.6	4.7	216	203
5	M	57	8.3	5.0	430	301
6	M	57	6.7	4.8	328	253
7	M	65	7.2	4.8	375	230
8	M	64	7.2	4.6	327	296
9	M	55	6.9	4.6	300	211
10	M	71	7.1	4.7	290	267
11	F	64	8.1	5.4	293	192
12	M	71	7.2	4.5	233	213
13	M	55	7.2	4.5	365	227
14	M	68	7.4	4.4	300	255
15	F	67	7.0	4.5	279	172
16	F	71	7.0	4.5	297	220
17	M	60	6.7	4.2	284	183
18	M	61	7.0	4.7	260	198
19	M	55	6.4	3.9	304	176
20	F	70	7.6	4.7	398	225
21	M	70	7.3	4.6	257	250
22	F	67	7.4	4.5	304	194
23	M	60	7.0	4.4	338	234
24	F	77	8.1	5.0	416	274
25	F	62	7.5	4.6	284	169
26	F	64	7.2	4.5	280	208
27	M	65	7.4	4.6	264	231
28	M	61	7.8	4.3	319	239
29	M	61	7.3	4.6	309	271
30	M	66	7.5	5.0	375	278
31	F	65	7.6	4.5	339	288
32	M	73	7.1	4.4	306	211

Ave ± SD		64.4 ± 5.7	7.3 ± 0.4	4.6 ± 0.3	317 ± 53.4	230.3 ± 36.9

M: male, F: female. TP: total protein. ALB: albumin. Che: cholinesterase. T-Cho: total cholesterol. Ave: average. SD: standard deviation.

From number 1 to 4 were used for 2-0 E, from number 1 to 8 were for first western blot, from number 9 to 32 were for second western blot.

**Table 2a:** (a) Proteins upregulated in pancreatic cancer.

Protein's name	Experimental	Theoretical	Score^(1)^	Queries	Validation
	mass (Da)	mass (Da)		matched^(2)^	
Histidine-rich glycoprotein precursor	80000	59541	150	3	WB^(3)^
Plasminogen precursor	100000	90510	331	8	
IGHM protein	50000	52754	105	2	
TF Serotransferrin precursor	75000	77000	601	14	WB
Isoform LMW of Kininogen-1 precursor	70000	47853	242	6	
F2 Prothrombin precursor (Fragment)	90000	69992	486	8	
Alpha-1B-glycoprotein precursor	43000	54239	590	8	WB
Vitronectin precursor	62000	54271	581	8	WB
Hepatocyte growth factor-like protein precursor	85000	80268	284	6	WB
Plasma kallikrein precursor	90000	71323	246	8	
Ceruloplasmin precursor	115000	122128	1452	49	
Isoform 1 of Ficolin-3 precursor	34400	32883	58	1	
Transthyretin precursor	95000	15877	4489	13	
Serum amyloid P-component precursor	24000	25371	2293	12	
Antithrombin III variant	61000	52658	1984	20	
SERPINC1 protein	61000	29074	1147	11	
Carbonic anhydrase 1	30800	28852	679	10	WB
Isoform 1 of C-reactive protein precursor	26000	25023	169	6	
Apolipoprotein A-1	31600	30759	225	4	
Isoform 1 of Inter-alpha-trypsin inhibitor heavy chain H3	138000	75031	529	12	
Leucine-rich alpha-2-glycoprotein precursor	47000	38154	549	8	
Isoform 1 of N-acetylmuramoyl-L-alanine amidase precursor	68900	67957	193	4	
Xaa-Pro dipeptidase	53700	54513	144	3	
Inter-alpha (globulin) inhibitor H4	130500	103261	1242	26	WB
Vitamin K-dependent protein S precursor	84000	75074	136	4	WB
Serpin peptidase inhibitor, clade D (Heparin cofactor),	72000	57034	374	9	
Isoform 1 of Fibronectin precursor	200000	262442	553	14	WB
Alpha-1-antichymotrypsin precursor	62800	50566	3315	33	WB
Kallistatin precursor	59600	48511	123	2	
Plastin-2	72000	70245	215	5	
Corticosteroid-binding globulin precursor	62500	45112	52	1	
Myosin-1	31600	222976	96	2	
Isoform 1 of Serum albumin precursor	73000	69321	261	9	
Cholinesterase precursor	83000	72836	50	1	
AMBP protein precursor	200000	38974	68	1	
Plasma protease C1 inhibitor precursor	87000	55119	1168	15	
Apolipoprotein B-100 precursor	300000	515241	3982	117	

^(1)^MOWSE score of candidate proteins.

^(2)^Number of peptide fragments yielding informative MS/MS.

^(3)^WB: western blot.

**Table 2b:** (b) Proteins downregulated in pancreatic cancer.

Protein's name	Experimental	Theoretical	Score^(1)^	Queries	Validation
	mass (Da)	mass (Da)		matched^(2)^	

Plasma retinol-binding protein precursor	19000	22995	373	8	WB^(3)^
Coagulation factor XII precursor	75000	67774	140	5	
Tetranectin precursor	19000	22552	61	1	WB
Hyaluronan-binding protein 2 precursor	68000	62630	195	6	
Vitamin D-binding protein precursor	55000	52883	284	14	WB
Hemopexin precursor	75000	51643	635	8	WB
Lumican precursor	100000	38405	120	6	WB
Isoform 1 of Gelsolin precursor	80000	85644	1360	23	WB
Afamin precursor	80000	69024	307	7	
Carboxypeptidase N catalytic chain precursor	49000	52253	244	12	WB
Inter-alpha-trypsin inhibitor heavy chain H1 precursor	200000	101326	812	13	WB
Histone H4	25700	11360	59	1	
JUP JUP protein	100000	81675	90	3	
apolipoprotein A-IV precursor	42000	45371	2188	35	WB
Inter-alpha-trypsin inhibitor heavy chain H2 precursor	200000	106370	1636	26	WB
Pigment epithelium-derived factor precursor	50000	46313	529	11	
Angiotensinogen precursor	56300	53121	986	13	
SERPINF2 protein	58000	55029	75	2	
Actin, cytoplasmic 1	100000	41710	208	5	
Thrombospondin-1 precursor	175000	129300	152	4	
Alpha-2-macroglobulin precursor	180000	163175	559	18	

^(1)^MOWSE score of candidate proteins.

^(2)^Number of peptide fragments yielding informative MS/MS data. The minimum significant threshold level of the probability-based MASCOT/MOWSE score was set at 5%.

^(3)^WB: western blot.

**Table 3a:** (a) Serum tumor marker levels in patients with pancreatic cancer.

Gender	Age (years)	UICC-stage	Tumor size (mm)	CA19-9 (U/mL)	CEA (ng/mL)	ApoA-IV (AU)	GC (AU)	RBP4 (AU)
M	38	IA	10	26.2	2.3	262.1	35.9	54.2
M	50	IB	30	46.5	2.3	8.5	34.3	43.9
M	63	IIA	18	157	1.1	10.2	26.7	37.0
M*	62	IIA	38	9	5	70.7	25.1	41.5
M*	54	IIA	24	11	1.4	87.5	11.3	42.5
M	73	IIA	25	15.5	3.5	203.5	13.6	49.5
F*	76	IIA	26	13.3	1.5	70.7	33.1	43.2
M	65	IIB	32	1579	3.4	352.1	24.3	36.7
M*	74	IIB	27	10.9	3.4	0	28.0	35.3
F	74	IIB	40	43	3.2	54	25.8	38.9
F	68	IIB	27	302	2.1	82.8	40.8	36.4
M	61	IIB	30	10	1.4	134.7	27.3	46.2
M	63	IIB	15	1080	—	144.6	23.9	53.3
M*	73	IIB	50	13.9	2.1	23.2	1.8	43.1
F	62	IIB	25	11.5	1.1	111.7	28.4	43.1

Ave ± SD	63.7 ± 1 0.4		27.8 ± 9.9	221.9 ± 466.2	63.7 ± 10.4	107.8 ± 99.9	25.4 ± 10	50.2 ± 4.2

CA19-9: carbohydrate antigen, CEA: carcinoembryonic antigen, ApoA-IV: apolipoprotein A-IV, GC: vitamin D-binding protein, RBP4: plasma retinol binding protein 4.

The stars indicate the patient who had a normal CA19-9 level and a low ApoA-IV.

Ave: average. SD: standard deviation. AU: arbitrary unit.

**Table 3b:** (b) Serum tumor marker levels in healthy controls.

Gender	Age (years)	CA19-9 (u/mL)	CEA (ng/mL)	ApoA-IV (AU)	GC (AU)	RBP4 (AU)
M	71	33.9	2.2	157.4	29.5	47.7
M	55	0.1	4.7	243.8	35.0	48.5
M	55	6.4	1	223.0	35.3	54.3
M	55	0.1	3.5	237.1	33.0	48.1
M	60	7.7	0.8	237.1	23.9	44.1
M	60	37.4	7	214.0	39.6	48.8
M	61	7.6	4.8	277.0	16.8	49.7
F	77	7.1	1.1	294.5	24.2	43.7
M	61	7.8	1.3	80.6	33.4	55.3
F	61	6	3	210.1	45.8	56.3
F	65	2.9	2.1	65.7	61.7	53.3
M	66	5.1	0.7	156.7	33.8	44.7
F	62	11.7	1.7	122.9	33.0	50.9
F	64	8.4	1.2	222.9	38.7	50.3
F	71	24.4	2.6	184.7	30.5	56.3

Ave ± SD	62.9 ± 6.3	11.1 ± 11.5	2.5 ± 1.8	195.2 ± 66.9	34.3 ± 10.3	50.2 ± 4.2

CA19-9: carbohydrate antigen, CEA: carcinoembryonic antigen, ApoA-IV: apolipoprotein A-IV, GC: vitamin D-binding protien, RBP4: plasma retinol binding protein

Ave: average. SD: standard deviation. AU: arbitrary unit.
