# Supplementary material for: The Application of a Three-Step Proteome Analysis for Identification of New Biomarkers of Pancreatic Cancer
Source: Int J Proteomics. 2011 Oct 17;2011:628787. doi: 10.1155/2011/628787 (PMC3199071; doi:10.1155/2011/628787)

Supplemental Figures:

Supplemental figure a. Raw data of Isoform 1 of ficolin-3 precursor

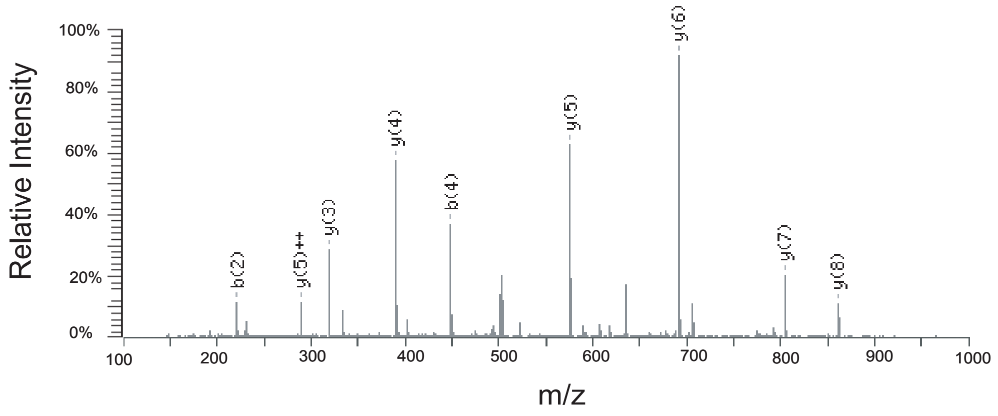

Supplemental Figure b. Raw data Corticosteroid-binding globulin precursor

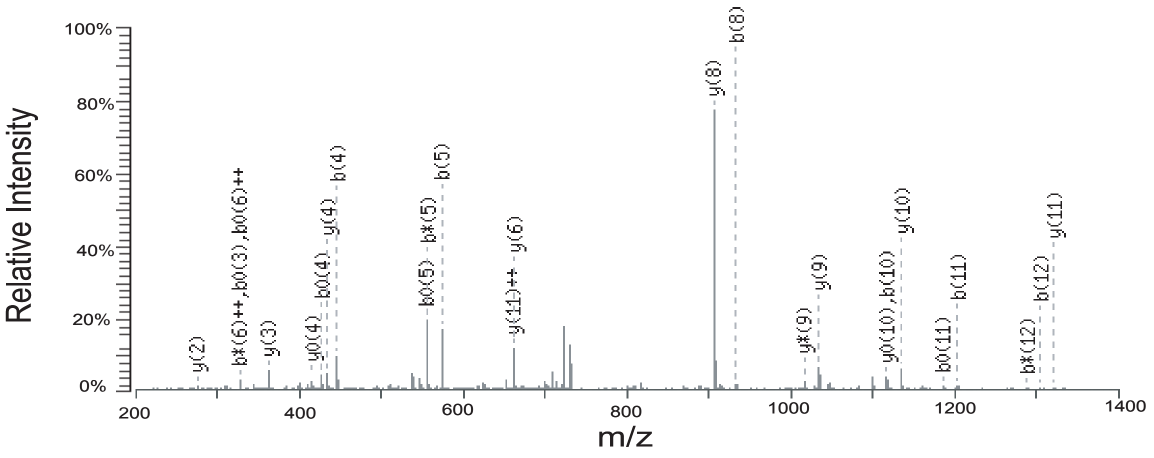

Supplemental Figure c.Raw data of Cholinesterase precursor

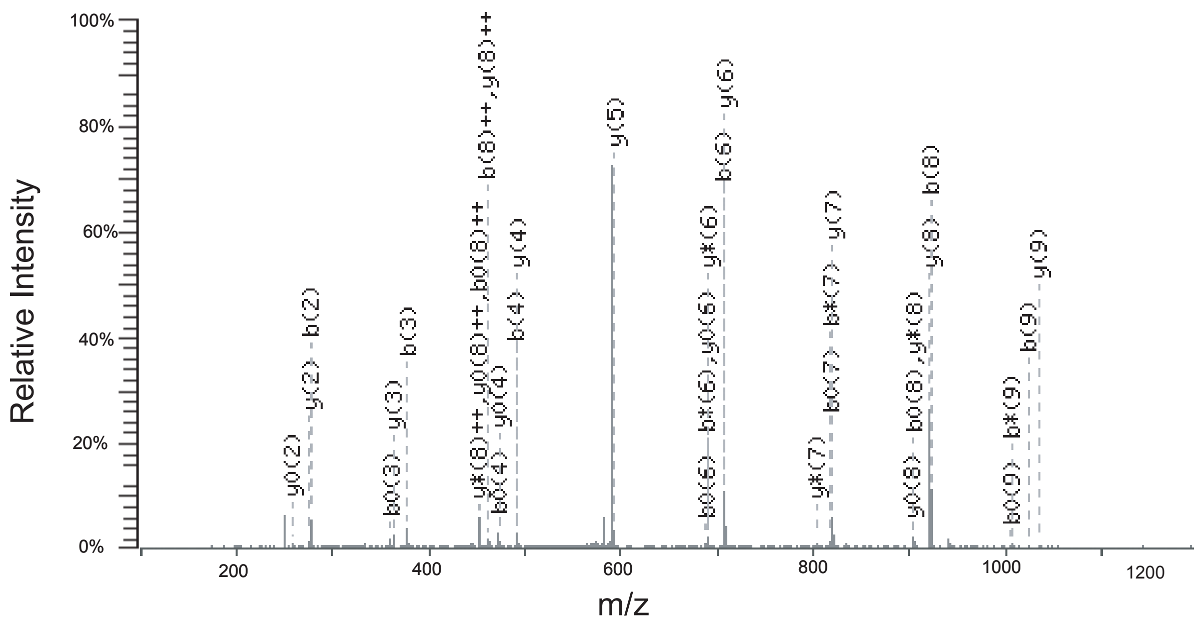

Supplemental Figure d. Raw data of AMBP protein precursor

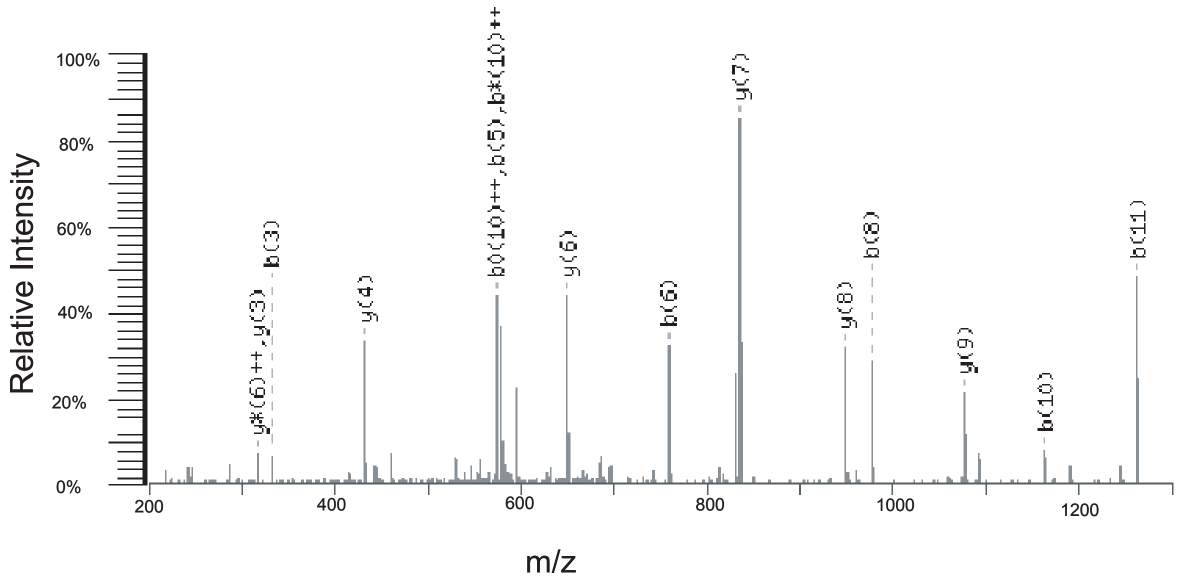

Supplemental Figure e. Raw data of Tetranectin precursor

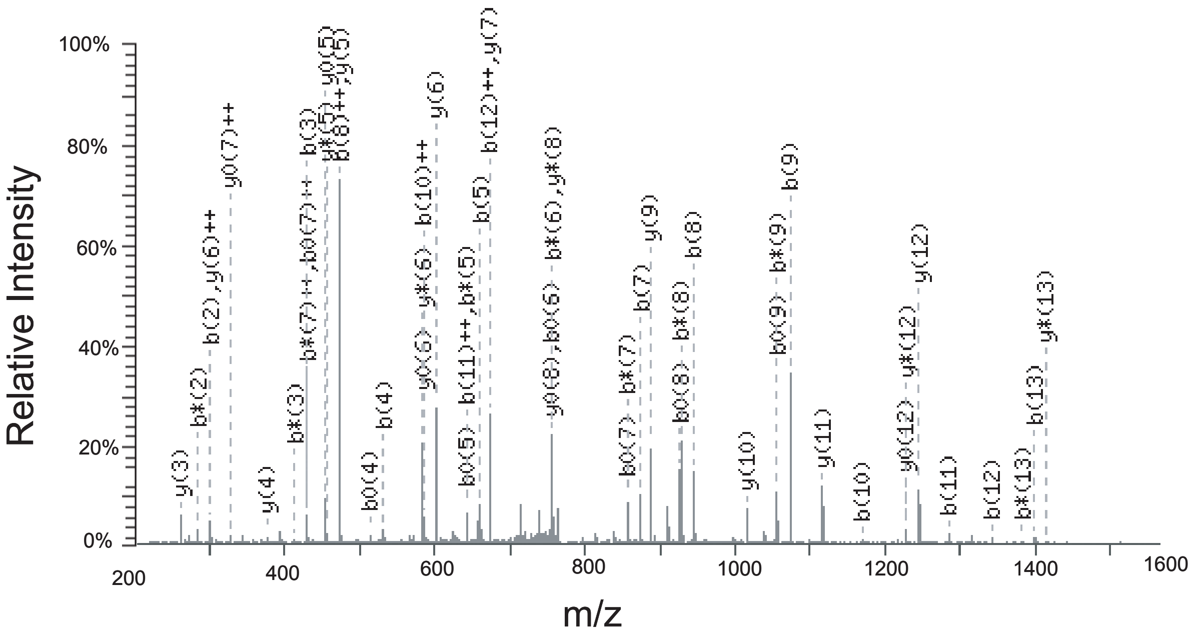

Supplemental Figure f. Raw data of Histone 4

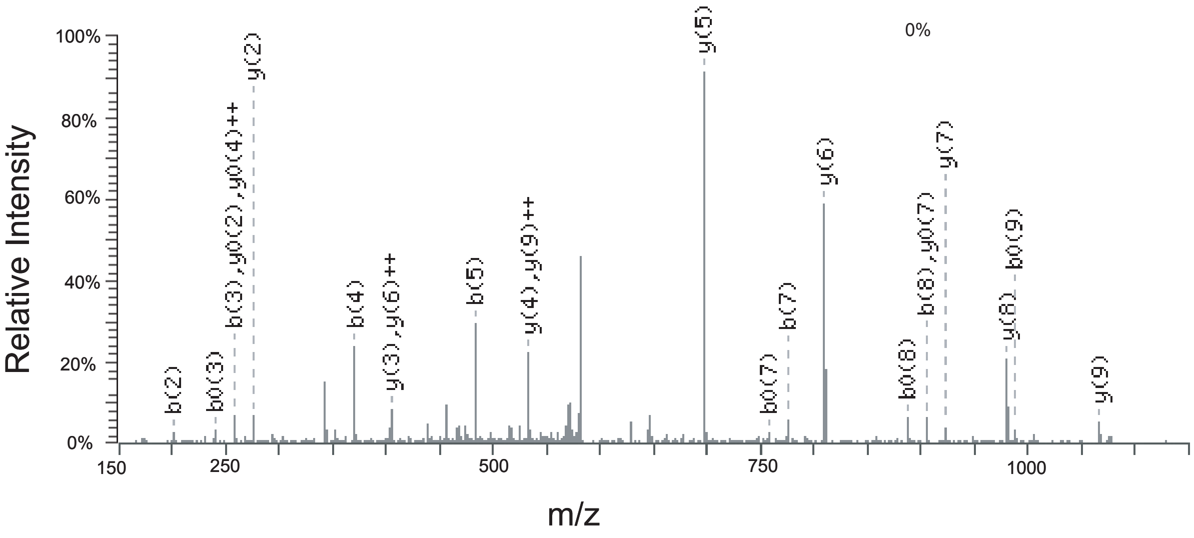

Supplement: Supplementary file 1 — Supplemental Figures: Raw data of the proteins which had SEQUEST scores lower than 100 or when the SEQEUST score was computed by using fever than one peptides fragment. The horizontal axis is molecular weight of peptide and the y-axis is intensity. Figure a: Raw date of isoform 1 of flcolln-3-precursor. Figure b: corticosteroid-binding globulin precursor. Figure c: cholinesterase precursor. Figure d: AMBP protein precursor. Figure e: tetranectin precursor. Figure f: histone 4. [file 628787.f1.pdf]
